# A cross-cultural study of unwillingness to consume insects in Croatia, Lithuania, Portugal, Romania, and Mexico

**DOI:** 10.3389/fnut.2025.1699378

**Published:** 2025-12-08

**Authors:** Rosa María Mariscal-Moreno, Anayansi Escalante-Aburto, César Ozuna, Raquel Guiné, Cristina Chuck-Hernández, Marijana Matek Sarić, Elena Bartkiene, Monica Tarcea, Lucio Rodríguez-Sifuentes

**Affiliations:** 1 Departamento de Salud, Universidad Iberoamericana, Ciudad de Mexico, Mexico. Prol. Paseo de la Reforma 880, A. Obregon Mexico C. P, Mexico; 2 Tecnologico de Monterrey, Institute for Obesity Research, Monterrey, Nuevo Leon, Mexico; 3 Departamento de Alimentos, División de Ciencias de la Vida, Campus Irapuato-Salamanca, Universidad de Guanajuato, Irapuato, Mexico; 4 CERNAS-IPV Research Centre, Polytechnic Institute of Viseu, Campus Politécnico, Portugal; 5 Department of Health Studies, University of Zadar, Zadar, Croatia; 6 Department of Food Safety and Quality, Institute of Animal Rearing Technologies, Lithuanian University of Health Sciences, Kaunas, Lithuania; 7 Department of Community Nutrition and Food Safety, University of Medicine, Pharmacy, Science and Technology, Targu-Mures, Romania; 8 Laboratorio de Biotecnología e Innovación, Facultad de Ciencias Biológicas, Universidad Autónoma de Coahuila, Torreón Coahuila, Mexico

**Keywords:** entomophagy, high nutritional value, insect-based product, low environmental impact, food neophobia

## Abstract

**Introduction:**

Insects are a nutritious and environmentally sustainable alternative to traditional animal proteins. Because of this, their use as human food is being implemented in Europe, although this practice is not well-received in the countries of that continent. On the other hand, there are countries like Mexico, with a long tradition of insect consumption, which can serve as a model for increasing insect consumption in European countries.

**Methods:**

A survey on insect consumption was conducted in Mexico, Croatia, Lithuania, Portugal, and Romania, and focused on people who had never consumed insects. The Chi-square test was performed for demographic variables; multinomial logistic regressions were used to establish relations between sociodemographic variables and respondents who had not consumed insects. Data from surveyed individuals from Mexico who have not included insects in their diet were analyzed to propose strategies to boost insect consumption in European countries.

**Results and discussion:**

The percentage of respondents who had not consumed insects in Mexico was 29.7%, whereas in European countries, it ranged from 70.5 to 89.3%. The Chi-square test revealed a statistically significant difference for all analyzed sociodemographic factors. Gender, age, educational level, geographical area, and income all influenced a person to be a non-consumer of insects. We suggest using isolated insect protein or insect flour to formulate/develop food products, inform about the nutritional and health benefits of insects, integrate insects into the countries’ traditional cuisine, and reduce the costs of insects to increase their consumption in European countries.

## 1 Introduction

The increase in the world population has brought the need to produce nutritious and healthy food with a lower environmental impact on the planet. Edible Insects are an excellent source of human food since they contain a high amount of high-quality proteins (40–75%), minerals (3–8%), fatty acids (7–77%), fiber (2–16%), and vitamins such as retinol, riboflavin, pantothenic acid, biotin, and folic acid (1). Additionally, insect production represents a much more sustainable way to produce protein for human consumption compared to livestock production (2).

However, some European countries are unfamiliar with consuming insects despite the nutritional and sustainable advantages of incorporating insects into the human diet. Significant aspects of culinary tradition, environment, history, community dynamics, human activities, mobility, and economic status of each region also influence this pattern (3), as well as the individual perceptions (4).

We conducted an electronic survey in Portugal, Croatia, Romania, and Lithuania, European countries without traditional practices of entomophagy. Mexico was also included, given its cultural history of insect consumption, to identify potential barriers or cultural influences in low-consumption geographical areas. Additionally, demographic data were collected from all countries in this survey. Portugal was selected, representing Southwest Europe; Croatia, representing Central and Southern Europe; Romania, representing Southeast Europe; and Lithuania, representing Northern Europe. These countries exhibit cultural differences in insect consumption compared to Mexico.

Dislike and repulsion toward eating insects can be attributed to neophobia, the fear of trying new foods. In the case of Europe, Statista reported that 83 and 71.5% of the population in Lithuania and Portugal, respectively, are not willing to replace meat with insects and insect derivates (5). In the case of Romania, recent studies highlight that the level of knowledge about edible insects is broad in this country. Some of the population consider them a great source of nutrition, particularly protein, while others believed that insect consumption can be dangerous for their health (6).

Additionally, in recent years, surveys have been carried out in different countries from Europe to assess the population’s acceptance toward the consumption of insects and to understand the barriers preventing a higher intake of this protein source. Grasso et al. (7) found that people aged 65 and above in The United Kingdom, the Netherlands, Poland, Spain, and Finland showed a greater preference for traditional protein sources such as dairy, meat, and seafood, compared to alternative options like plant-based protein, single-cell protein, insect-based protein, and *in vitro* meat-based protein. Regarding insect consumption, only 9% of the respondents found it acceptable or very acceptable, while 91% answered with neutrality or found it unacceptable, very unacceptable, or expressed uncertainty. Piha et al. (8) found that individuals from Northern Europe (represented by Finland and Sweden) exhibited a more positive attitude toward consuming insects compared to their counterparts from Central Europe (represented by Germany and The Czech Republic). On a scale of 1–7, the willingness to purchase insect-based products was 3.3 in Northern Europe and 2.7 in Central Europe. However, we can generally conclude that insect consumption is not widely accepted in these European regions. In a survey across 13 countries on five continents involving individuals aged 18 to over 55, 61.53% of the countries expressed unwillingness to try insect-based products from a well-known company. The three European countries surveyed (The United Kingdom, Russia, and Spain) were part of this group, and their primary reasons for not consuming insects were: (1) I do not want insect pieces in my foods, (2) just the thought makes me sick, and (3) the idea is disgusting (9).

Another factor that can affect the consumption of insects in each region is regulations; for example, in European countries, the commercialization of insects is limited to specific species approved by the European Food Safety and Authority (EFSA) under the Novel Food Regulation. The number of insects approved by EFSA is limited, and points of sale are minimal (10).

In contrast, some countries exhibit strong entomophagy (habitual insect consumption as food) or positive attitudes toward insect consumption. As previously reported by Escalante-Aburto et al. (11), there is a long-standing tradition of insect consumption in Mexico; in their study, 74% of the Mexican population surveyed acknowledged having consumed insects. In a global survey, Castro and Chambers (9) reported that individuals from Mexico, Peru, Thailand, China, and Brazil expressed a willingness to consume insect-based products. Notably, in the case of Mexico, 71% of its population endorsed this idea, marking the highest score in the study. Meanwhile, 21% were reluctant to try insect-based products, and 8% remained unsure. The tradition of consuming insects in Mexican cuisine, as highlighted by Youssef and Spence (12), might be a critical factor in why the people of Mexico are familiar with entomophagy and do not express aversion toward this practice, as noted by Castro and Chambers (9).

We propose this study to identify and understand the factors contributing to the reluctance of non-insect consumers. The inclusion of a country like México will help support a shift in insect consumption patterns in countries without traditional insect-eating and explore strategies to encourage the consumption of insect-based foods.

## 2 Methods

### 2.1 Study information

The study focused on individuals who have never consumed insects in their lives, aiming to correlate them based on demographic data, comprehend their opinions about this practice, and propose strategies to promote entomophagy in these countries. Furthermore, the same survey was carried out in Mexico to draw comparisons between European countries, where insect consumption is not typical, and countries with a tradition of consuming insects. A total of 527, 686, 492, 510, and 1,139 individuals (*n* = 3,354) aged 18 and over were anonymously surveyed in Portugal, Croatia, Romania, Lithuania, and Mexico, respectively. All participants provided consent for the use and publication of the collected data.

In our previous study (11); we surveyed 3,125 Mexicans, of whom 811 had not consumed insects. These unpublished data were also considered. The analysis focused on the reasons for their avoidance of insects in their diet and the factors that could justify incorporating this protein source into their diet.

### 2.2 Survey design

The questionnaire comprised two questions along with demographic information about the participants. Initially, respondents were asked, “*Have you ever consumed insects as culinary preparations, snacks, or other derived products*?” Those who answered “*Yes*” or “*Do not know/Do not remember*” were excluded from the study, while those who answered “*No*” were included in the analysis of their responses regarding sociodemographic information. Additionally, they were asked to provide up to five words or short phrases associated with edible insects.

The survey was translated into Portuguese, Croatian, Romanian, Lithuanian, and Spanish and then shared through a link on the social media platforms of each respective country. Please Refer to Supplementary Tables 1–8 for the translated version. The questions were shared through the Google Forms® platform from July 2021 to November 2021. The questionnaire for Mexico was answered by voluntary and anonymous individuals, as required by Mexican law NOM-012-SSA3-2012 (13). Participants were informed that the collected data would be published in a journal article and would remain confidential and anonymous. For Portugal, Croatia, Romania, and Lithuania, the questionnaire received approval from the Ethics Committee of the Polytechnic University of Viseu (45/SUB/2021).

### 2.3 Data analysis

For the question: *Have you ever eaten insects as culinary preparations, as snacks, or other derived products*? The percentages of participants who selected one of the three possible answers provided were calculated.

A descriptive analysis was conducted on the sociodemographic variables (age, gender, educational level, geographical area, and income) within the study sample. Categorical variables were presented as frequencies and proportions. We used the Chi-squared test to determine significant differences in sociodemographic variables between participants who reported not consuming insects across all study countries and within each country, with a *p* < 0.05 Multinomial logistic regression models were employed for estimation to examine the correlation between the non-consumption of insects and the sociodemographic variables of interest. Because all participants answered the same questionnaire and the analysis categories were equivalent, a combined analysis was conducted to identify general or global patterns from the data obtained in the five countries. All analyses were conducted using STATA 15 statistical software (StataCorp LLC, College Station, TX, USA).

Non-consumer individuals were requested to share the words that come to mind when they hear about edible insects. Participants were allowed to submit up to five terms. The list of words provided by the participants was carefully evaluated, and similar expressions or synonyms were grouped into overarching word categories. For example, the words *“Thailand,” “China,” “Japan,” “India,” “Asia,” “Africa,”* and other similar terms were grouped under the expression *“Consumption-countries.”* Similarly, the words and expressions *“Disgusting,” “Disgust,” “Disgusting food,”* and *“That they are disgusting”* were grouped under the word “Disgusting” in the case of Mexico. The repetition percentage was calculated by Equation 1. This method accurately indicates the extent of word repetition in this question.


(1) $$\begin{array}{l} \%\text{Repetition}=\\ \;\frac{\text{Number of times}\;a\;\text{word was repeated}}{\text{Total number of obsevations gathered from the survey}}\times 100 \end{array}$$

In our previous study, we explored the questions: “Of the following reasons, which would justify why you do not include insects in your food/diet?” and “Of the following reasons, which would justify including insects as part of your nourishment/diet?” we presented 9 and 10 options, respectively, and asked the respondents to select all that applied. The percentage of times an option was determined relative to the total number of responses was calculated.

## 3 Results

### 3.1 Insect consumption

**Figure 1:**
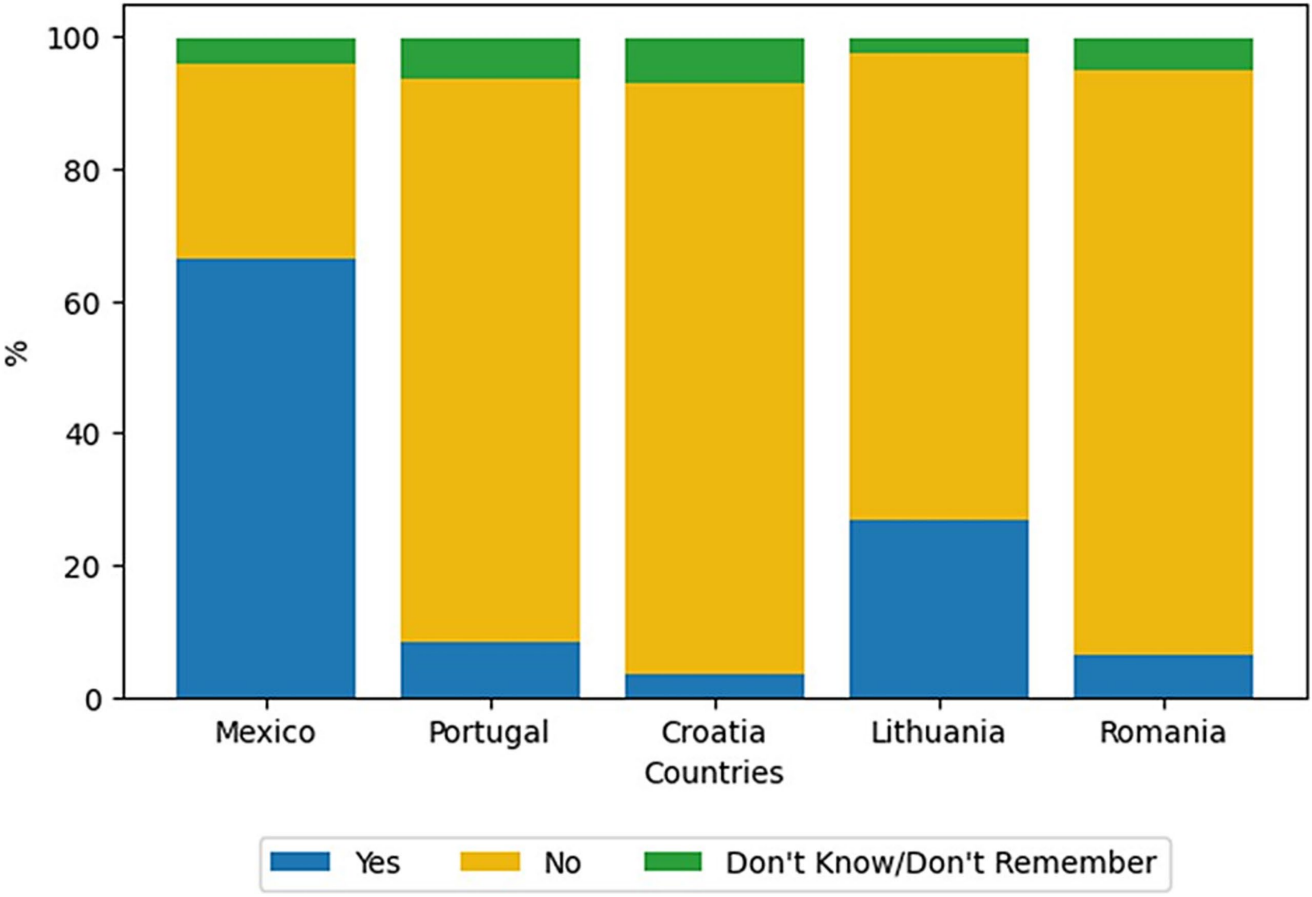
Percentage of participants who answered “No,” “Yes,” or “Do not know/Do not remember” to the question: “*Have you ever eaten insects as culinary preparations, as snacks, or other derived products*?”

In the primary survey, the question *Have you ever eaten insects as culinary preparations, as snacks, or other derived products?* With the possible answers “No,” “Yes,” or “Do not know/Do not remember” aimed to assess insect consumption in the countries under scrutiny in this research. As depicted in Figure 1, in the case of Mexico, in contrast to European countries, most of the population has embraced insect consumption, with 66% of the respondents in this country having reported doing so. In contrast, the surveyed populations from Lithuania, Croatia, Portugal, and Romania reported an average of 17% respondents stating that they do consume insects. Interestingly, in Portugal and Romania, the percentage of respondents who have consumed and the consumers in the same country who answered “do not know/do not remember” are relatively similar.

### 3.2 Demographic data

The sociodemographic factors considered were gender, age group, educational level, geographical area, and income. Table 1 represents the analysis of sociodemographic factors among individuals within the sample group (*n* = 2,197 who do not consume insects), categorized by country. Chi-square test results indicate statistically significant differences in sociodemographic values (*p* ≤ 0.05). The odds ratios are shown in Table 2, which presents the relative odds of the first category within each group compared to the other classes within the same demographic group.

**Table 1:** Sociodemographic characteristics among non-consumer insects from the five countries evaluated (*n* = 2,197).

Demographic characteristic	Percentage (%)					*p* value*
	Mexico	Croatia	Lithuania	Portugal	Romania	
	*n* = 339	*n* = 613	*n* = 360	*n* = 449	*n* = 436	
Gender						
Male	47	43	41	25	11	0.000
Female	52	57	58	75	89	
No answer	0	0	1	0	0	
Age group						
18–30 years	70	42	58	53	20	0.000
31–50 years	27	36	36	24	60	
51+ years	3	22	20	24	20	
Educational level						
No university degree	55	66	35	48	13	0.000
Completed a university degree	37	31	34	22	37	
Post-graduate education (master or doctorate)	8	3	31	30	50	
Geographical area						
Rural	8	20	5	32	12	0.000
Suburban	12	19	30	12	4	
Urban	79	61	66	56	83	
Income						
Much higher	6	3	9	11	8	0.000
Higher	28	24	26	25	49	
Equal to average	17	57	50	39	33	
Lower	31	15	9	16	10	
Much lower	18	2	5	8	1	

*p-values were calculated using the chi^2^ test. Values denote statistical significance at *p* < 0.05.

**Table 2:** Logistic regression analysis to estimate the probability of non-consumption based on sociodemographic characteristics among the five studied countries (*n* = 3,176).

Demographic characteristics	All countries		
	Odds Ratio	CI (95%)	*p*-value
Gender			
Female	Ref		
Male	2.64	2.26–3.07	0.000
Not answer	5.50	1.79–16.91	0.003
Age group			
18–30 years	Ref		
31–50 years	0.88	0.75–1.03	0.135
+51 years	0.34	0.25–0.45	0.000
Educational level			
No university degree	Ref		
Completed a university degree	1.64	1.38–1.93	0.000
Post-graduate education (master or doctorate)	1.01	0.82–1.23	0.923
Geographical area			
Rural	Ref		
Suburban	3.06	2.22–4.22	0.000
Urban	2.86	2.15–3.77	0.000
Income			
Equal to average	Ref		
Higher	1.77	1.45–2.15	0.000
Lower	2.59	2.07–3.22	0.000
Much higher	2.38	1.76–3.19	0.000
Much lower	3.32	2.49–4.41	0.000

CI 95%, Confidence Interval of 95%.

The odds ratio was determined from multinomial logistic regressions.

In Portugal and Romania, most respondents were women (73.86 and 88.94%, respectively). In Croatia, Lithuania, and Mexico, the gender distribution was reasonably balanced, with approximately 50% of respondents being male and 50% female (Table 1). The odds ratio of 2.64 for males indicates that they are 2.64 times more likely to be no-consumers than females (Table 2).

Most of the data obtained were from individuals aged 18–30 years, except for Romania, where most respondents who had not consumed insects belonged to the 31–50 age group. In all surveyed countries, the minority population fell within the age range of 51 years and older, except for Portugal (Table 1). The reference age group was 18–30 years, which was used to calculate the odds ratio. The 31–50 age group did not significantly differ (*p* = 0.135). However, the +51 age group showed a slightly higher probability of being non-consumers (Table 2).

Based on the education level of the respondents, it was found that most non-consumers did not have a university degree. It is worth noting that Croatia and Mexico had the lowest percentage of respondents with post-graduate education. In Lithuania, the distribution among the three assessed education levels was similar (Table 1). Compared to individuals who did not complete university, those with a university degree are more likely not to consume insects. However, this trend does not hold for post-graduate students, as it lacks statistical significance. Education seems to be a significant factor influencing the willingness to consume insects, but this will be discussed later in the discussion section (Table 2).

When the income variable was assessed, most respondents presented an income equal to the average, except for Mexico and Romania (Table 1). The most significant odds ratios for income are 3.32 for much lower income, 2.59 for lower income, 2.38 for much higher income, and 1.77 for higher income. These findings regarding income ranges suggest that contestants with lower incomes are more likely to be non-consumers, while the likelihood decreases for contestants with average income (Table 2).

**Figure 2:**
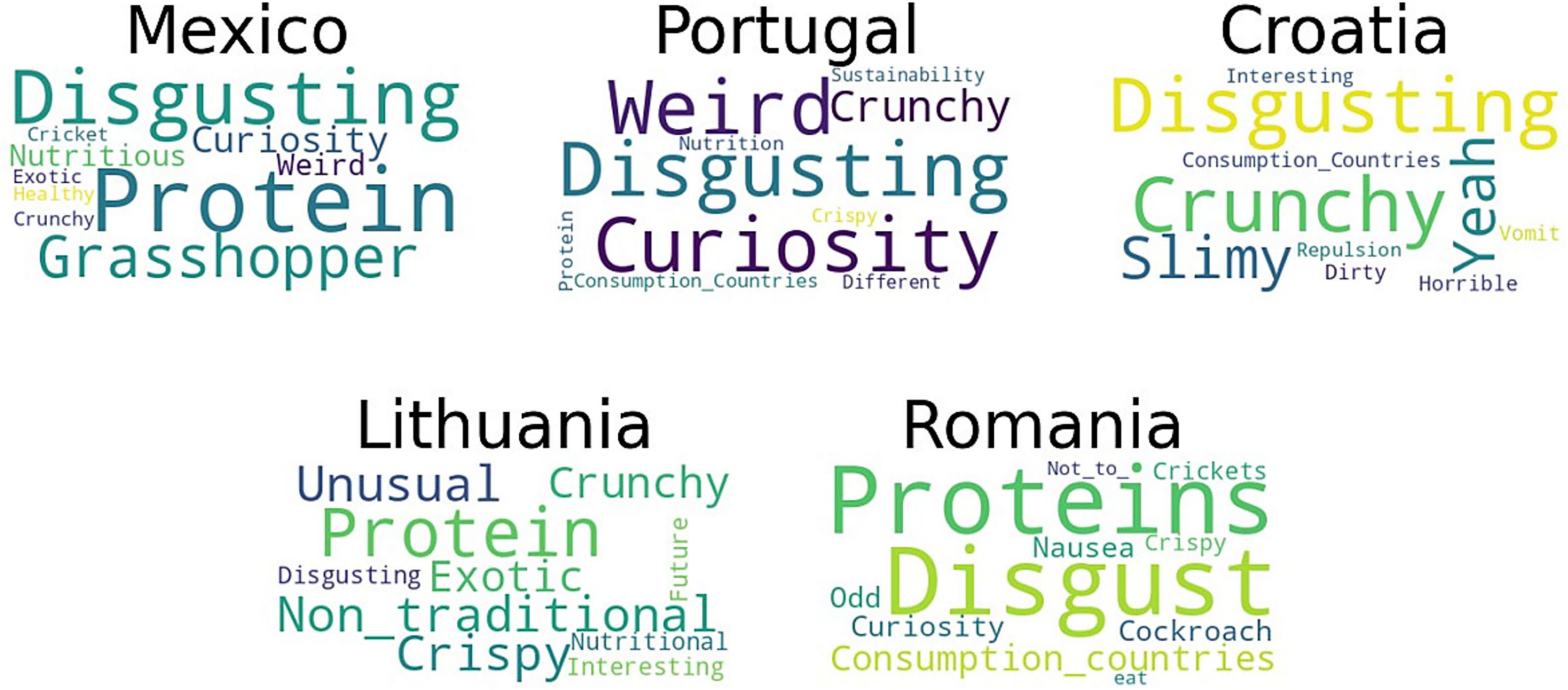
Word Clouds with the 10 most frequent words from participants who have not consumed insects from each country associate them when hearing about edible insects.

During the survey, participants who reported not consuming insects were asked to share their thoughts on the perception of edible insects. They were instructed to provide up to five keywords or expressions to help assess factors predicting their reluctance toward insect consumption. The responses from participants in five countries were analyzed to determine the most common characteristics, and the top 10 frequently mentioned words were used to create a word cloud (Figure 2). It is worth noting that the term “*disgusting*” was found to be more commonly associated with insect consumption in Portugal, Croatia, and Romania. This term reflects the emotional reactions of disgust that many people experience at the idea of eating insects. This disgust is based on cultural and personal associations that link insects with poor hygiene, disease, and unpleasant sensations related to sensory characteristics. Furthermore, among these countries, the most frequently occurring words primarily revolved around negative aspects, indicating feelings of disgust, such as weird, vomiting, nausea, and dirty. However, although less frequently, other words related to positive factors, like sustainability and nutrition, also appeared among the most used terms. On the other hand, in Lithuania and Mexico, the most frequently used word was “*protein*,” suggesting that people in these countries are more inclined to perceive insect consumption as a nutritional or alternative protein source. The positive factors obtained can be associated with the recent increase in the population’s interest in improving health through preventive measures, such as adopting a healthy and sustainable diet.

Notably, the cumulative percentage of the top 10-word percentages consistently exceeds 37% in all instances. In some cases, such as Romania, these percentages surpass 50%. This suggests that these terms represent the principal concepts associated with insects among non-consumers. They are the key aspects that must be addressed to increase insect consumption in countries without a traditional history of such practices. The word *“yeah”* is not Croatian, but it is used by younger people as the informal English equivalent of *“yes,”* the expression *“consumption-countries”* was used to refer to countries where the consumption of insects is common. Finally, as depicted in Figure 2, non-consumer participants in Portugal, Croatia, and Romania use the term “*consumption-countries*,” while Lithuania uses “*non-traditional*.” This suggests that these countries lack cultural appropriation of insect consumption and instead associate it with other countries and/or cultures.

**Figure 3:**
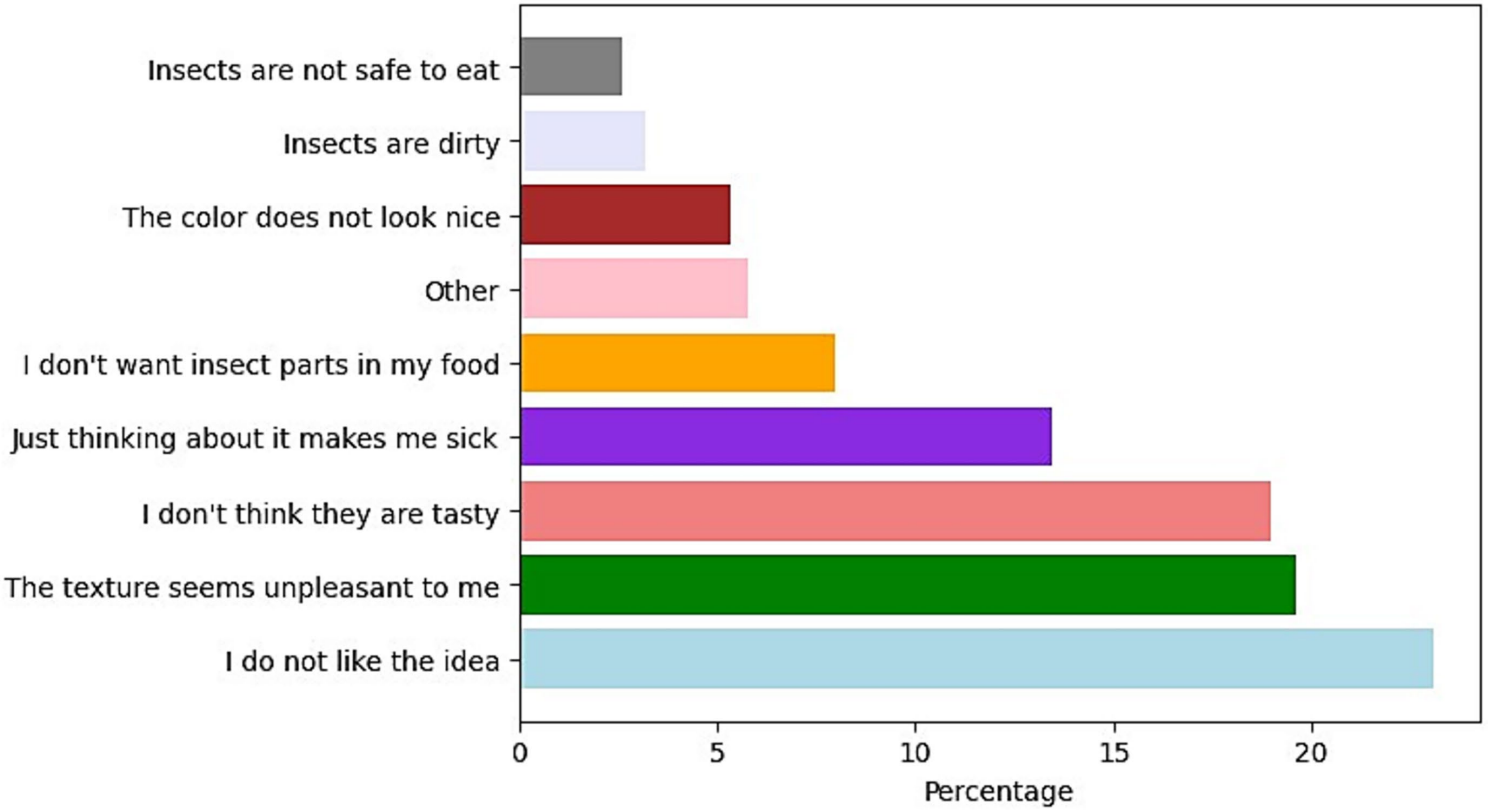
Percentage of mentions to the question “*Of the following reasons, which would justify why you do not include insects in your food/diet*?”

The unpublished responses to the questions applied to the Mexican population, which had not previously consumed insects in our prior study (11), were analyzed. This analysis aimed to clarify and propose actions to promote insect consumption in European countries and Mexico. Additionally, insects can fulfill sustainability attributes, promote healthiness, enhance accessibility, and provide tastiness (14). However, as discussed in previous sections, various factors can influence an individual’s willingness to consume insects. Figure 3 illustrates several of these factors, highlighting those that can be modified to enhance the willingness to consume insects.

The initial factor to consider is “I do not like the idea.” This serves as a broad concept that the subsequent elements aim to complement. About 52% of the responses are associated with sensory characteristics, with 20% focusing on texture, 19% on taste, 8% on the presence of insect parts in the food, and 5% on color.

**Figure 4:**
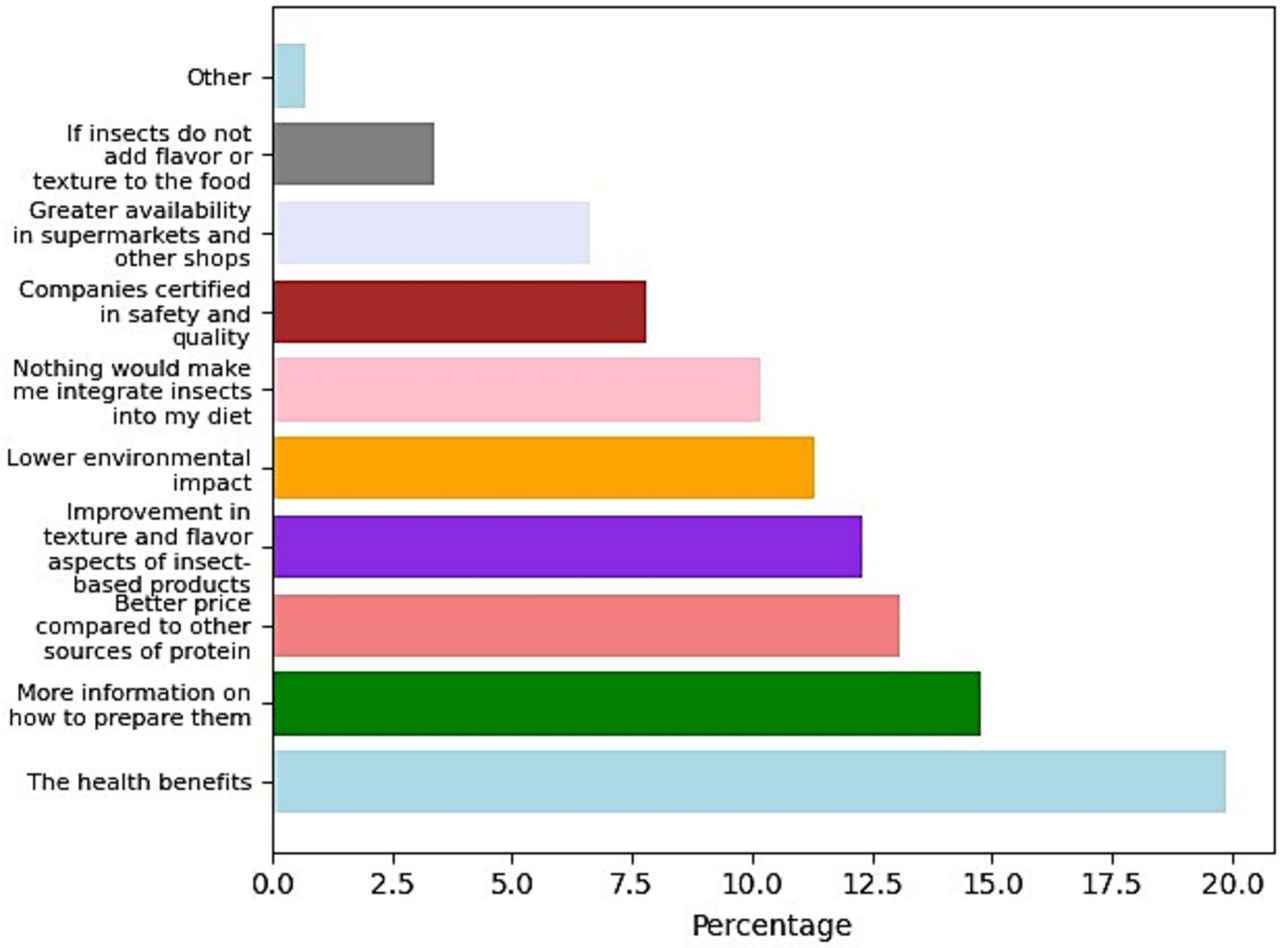
Percentage of mentions to the question “*Of the following reasons, which would justify including insects as part of your nourishment/diet?”*

Additionally, Figure 4 illustrates Mexican respondents’ increasing willingness to eat insects. The most frequent response was the health benefits (20%). Others stated that more information about preparation is needed (15%). Furthermore, an improvement in the price of insects compared to other protein sources (13%) would lead to increased consumption. Additionally, the study revealed that 12% of respondents are open to consuming more insects if there is an improvement in texture and flavor. Furthermore, 11% of individuals expressed willingness to increase their insect consumption if it contributes to a lower environmental impact. Finally, 8 and 7% identified safety and quality certification and availability in supermarkets and shops as factors that would boost insect consumption.

## 4 Discussion

As anticipated, Europeans exhibited limited experience in consuming insects (Figure 1) as they became acquainted with this alternative protein source (15). Mancini et al. (16) presented a review of articles published on the acceptability of edible insects in European countries, revealing that the potential of insects as food remains untapped mainly on the European continent, primarily due to a lack of knowledge about this food source. In a study conducted by Piha et al. (8), it was found that consumers in northern and central European countries exhibited little willingness to purchase products made from insects. However, consumers in Northern Europe displayed a more positive attitude toward insect consumption as food than those in Central Europe. In the present study, a similar behavior was observed when comparing participants from Lithuania, a Northern European country, with those from Croatia, a Central European country.

In the case of Mexico, the percentage of respondents who consumed insects (66%) was significantly higher than that in European countries (Figure 1). Castro and Chambers (9) conducted a global survey across 13 countries spanning five continents, revealing that 71% of Mexicans were willing to consume insect-based products. Mexico exhibited the highest percentage of the population disposed to eat this kind of product, followed by countries such as Peru (58%), Thailand (56%), Brazil (45%), and China (44%) (9). Bermúdez-Serrano (17) mentions that Mexico is a country with the habit of consuming insects due to its ethnic and biological diversity. This country has the highest number of recorded edible insect species in the world (545 species), and their consumption is not only for survival but also as a specialty in gastronomy (18).

According to a literature review conducted by Florença et al. (19), certain demographic factors can impact the acceptance of insect consumption, either positively or negatively. Most of the studies have reported that men are more willing to try and consume edible insects than women (19, 20). This pattern is attributed to lower neophobia and greater openness to novel food among males. Some exceptions have been observed depending on the contextual factors, such as culture, environmental concern, and education (19, 21). Similar to these exceptions, the present study found that women exhibited greater willingness to consume insect-based products compared to men (Tables 1, 2), suggesting that gender related patterns in entomophagy acceptance may be context-dependent and influenced by situational or sociocultural variables.

Florença et al. (19) conducted a systematic review, and they examine how age influences consumers’ willingness to eat edible insects across different cultural contexts. Their analysis indicated that age is a relevant but complex determinant of insect consumption. Among the 31 studies reviewed, 11 examined age as a factor, and eight identified it as a positive determinant influencing willingness to consume edible insects. In most cases, younger adults, typically below 35 or 54 years, showed higher openness toward entomophagy, particularly in Western populations where food neophobia tends to increase with age. One study did not specify a particular age group associated with greater acceptance. In accordance with these findings, our data revealed that participants over 51 years old were less likely to consume insect-based products (Table 2).

In this evaluation, participants with a university degree showed a higher likelihood of being non-consumers (Table 2). However, it is worth noting that previous studies suggested a different trend, indicating that individuals with a university degree were 8.37 times more likely to express interest in trying insects as a food source compared to those with a lower level of education (22). This led to the hypothesis that individuals with higher education and knowledge may exhibit a more positive attitude toward novelty than those with less education (20). However, an increase in knowledge alone does not necessarily lead to a significantly higher willingness to consume; underlying attitudes and cultural context remain decisive determinants (19).

As shown in Table 2, the trend in non-consumption is influenced by geographical area due to availability. In Mexico, individuals typically acquire edible insects for human consumption either from their relatives or by purchasing them at local markets and street vendors, particularly in the central and southern regions of the country (11). Additionally, some insect species, considered delicacies, are packaged and sold at high prices in markets and urban centers (23). Furthermore, Florença et al. (19) suggest that edible insect consumption is influenced by gender, age, cultural and traditional factors, food neophobia, feelings of disgust, and prior familiarity with these foods.

For the income variable, odds ratios indicate that individuals with lower incomes are more likely to be non-consumers, while consumption increases among those with average incomes. Although previous studies associated insect consumption with lower-income groups, their findings were mainly observed in rural areas, where entomophagy remains part of traditional diets (24). Additionally, Florenca et al. (19) reported that higher income was associated with greater knowledge and a higher likelihood of having tried edible insects, suggesting that income can influence consumption differently depending on the socioeconomic and cultural context.

Negative insect-associated factors have been previously identified, indicating a connection with specific sociodemographic characteristics such as age, gender, geographical area, occupation, and sensorial attributes (2). It is consistent with previous studies that have reported a strong aversion or disgust in Western societies without a cultural tradition of consuming insects when considering insects as potential additions to their diet (25).

Previous studies, such as the work by Palmieri et al. (40), have indicated that only a minority of participants considered positive health and environmental effects significant factors influencing their attitude toward alternative protein consumption. This aligns with the findings of our study, as illustrated in Figure 2. The sensory factor was substantial for the respondents, who associated insect consumption with countries with a tradition of eating insects, such as China, Thailand, Mexico, etc. (Figure 2). This association was prevalent in most of the evaluated countries. Insect consumption can be motivated by curiosity or similar factors. Curiosity can potentially help individuals explore novel experiences with insect-based food (26). In most European countries (except for Lithuania), the word “*disgusting*” was the most frequently associated term with the consumption of insects. Other negative words were also used to describe the perception in these countries, including *weird, vomiting, nausea, and dirty*. However, with less frequency, among the most frequently repeated words were those related to positive factors such as sustainability and nutrition (Figure 2).

The results of the survey conducted among the Mexican population that has not consumed insects (Figures 3, 4) help identify motivational factors that may be universal, such as the perception of insects as a sustainable protein source, curiosity about trying new foods, health-related benefits, and social influence. These elements, although observed in a Latin American context, can be adapted and leveraged in awareness campaigns across Europe, where entomophagy still faces significant cultural resistance. Furthermore, the Mexican case provides an opportunity to examine the dynamics of transition from rejection or indifference to a potential willingness to consume. This type of shift, studied in a setting where entomophagy is socially accepted, can serve as a model for understanding how similar behavioral changes might be facilitated in regions where insect consumption is not part of the traditional food culture. Finally, although some Mexicans do not eat insects, they live in an environment where insect-based products and recipes are present. This can provide ideas on how to integrate insects into traditional European dishes or into familiar formats such as cookies, energy bars, or snacks.

We conducted the following discussion to propose strategies to increase the preference for insect consumption in European countries. Sogari et al. (27) argued that the consumption of edible insects is primarily influenced by market factors such as regulation, framework, industry dynamics, and product-related aspects, including processing and familiarity. Effective communication and marketing also play crucial roles.

Creating a single proposal to enhance insect consumption is challenging due to the diverse factors influencing consumer acceptance, as evaluated in the results section. Nevertheless, specific strategies can be implemented throughout the food chain. In this context, some authors have proposed pretreatments or processes such as drying, defatting, protein extraction, and hydrolysis (25). However, evaluating alternative methods, such as non-thermal or green methods, is crucial to enhance the consumption of insects and improve the nutritional, sensory, and sustainable characteristics for human consumption.

To ensure the success of practical strategies aimed at boosting insect consumption, it is imperative to enhance the sensory properties of insects and insect-based foods, primarily focusing on improving factors such as texture, appearance, and taste (28).

Protein isolation from insects is a suggested solution to reduce negative sensory characteristics, allowing them to be used as a food ingredient. However, the parameters for protein isolation from insects are complicated because they depend on the chemical properties of each species, as well as the environmental conditions of cultivation and the feed employed to rear the insects. These parameters can modify specific composition characteristics such as lipid content, protein content, etc. (29). Moreover, the functional attributes of isolated proteins become crucial when considering their use as ingredients. Typically, a preference is given to proteins with high emulsification properties (30). However, there is a lack of comprehensive research on the functional attributes of isolated proteins, highlighting the need for further investigation to ascertain their potential applications in the food industry. According to Woolf et al. (31), the level of exposure to edible insects may affect consumers preference regarding the type of product. Consumers who have eaten insect-based foods are more willing to consume fried/grilled/roasted whole insects, while those who have never eaten insects are more willing to consume protein bars made with insect protein isolate.

Another option is to incorporate or substitute traditional ingredients with insects. Numerous suggestions have been made for utilizing insects as ingredients in the food industry. The most prevalent application involves integrating insects into the development of bakery and cereal-based products, primarily to enhance their nutritional characteristics. For example, de Oliveira et al. (32) utilized cinereous cockroaches to produce flour. The resulting flour exhibited a protein content of 63.22% and contained high-quality lipids. Subsequently, the flour was integrated into bread formulations at concentrations of 5, 10, and 15%. The findings revealed a remarkable 133% enhancement in protein content and a significant 64.53% reduction in fat. Other examples of application include the enhancement of snacks through 3D printing (33) and the incorporation of cricket powder into bread (34). Another compelling application, driven by the sustainability of the process, is the creation of non-meat products or the reduction of animal protein in food items. Hamburgers prepared with 50% mealworm larvae (*Tenebrio molitor*) have been developed. Recent reports indicate that companies are working on extracting and restructuring insect proteins to create meat substitutes as well as alternatives for eggs and dairy in food processing (35).

The consumption of insects should be promoted in European countries not only for their nutritional value (36) but also for their potential beneficial effects on human health. These include antioxidant, antihypertensive, anti-inflammatory, antimicrobial, and immunomodulatory activities (37).

Information on how to prepare insects was a crucial factor in incorporating them into the respondents’ diet. Knowledge of insect preparation recipes can boost their consumption in cultures where such types of food are not commonly shared. The importance of information and collaboration with culinary professionals to promote entomophagy was studied by Woolf et al. (31). In this study, an event was organized in The United States that included an educational session on insect consumption and a culinary demonstration with insect tasting. Attendees who completed surveys before and after the event reported feeling more informed and more willing to consume edible insects. Hurd et al. (18) mentioned the diverse dishes that can be prepared using insects in Oaxaca, Mexico. This information can serve as a foundation for insect preparation following the cultural practices of each country. The authors also discussed the processing methods for specific insects, such as *chapulines* and *cochineal* insects, aiming to prolong their shelf life and facilitate the creation of new insect-based products.

Currently, the price of insect protein is considerably higher than that of plant-based proteins and even higher than animal protein (38). However, considering that insects can convert organic matter into protein and oil, a proposal is to establish profitable insect farming for production purposes (39). Jensen et al. (39) also mentioned that, under this scheme, the growth of the insect-based food industry may contribute to a significant decrease in the price of oilseed and protein meals. Some limitations to suggest further research directions can be aligned to use a structured list of terms that consumers associate with insect consumption. This approach could reduce the number of categories and provide a more robust understanding of consumer perceptions. Additionally, future studies should examine the scalability and consumer acceptance of various processing methods for insect-based food ingredients. Such research would offer valuable insights into optimizing production processes and enhancing consumer acceptance.

## 5 Conclusion

Insect production for human consumption is a nutritious option with lower environmental impact compared to animal protein production. Despite these advantages, insect consumption has been poorly accepted in European countries. On the other hand, Mexico has a strong tradition of consuming this protein source, making it a reference for designing strategies to increase insect consumption in other countries.

This study revealed that sociodemographic factors influenced people from each country to avoid insect consumption. People from most European countries mentioned words or phrases denoting food neophobia toward insect consumption, while in Mexico insect consumption was associated with a nutritional source. Sensory characteristics played a significant role in participants’ perception of insect consumption. Regarding tendencies and perspectives, a significant aspect includes the development of processes to convert insects into food ingredients considering the aim to decrease the negative sensorial characteristics that non-consumers associate with insect consumption, thereby increasing the acceptability of insect food among the population.

## Data Availability

The raw data supporting the conclusions of this article will be made available by the authors without undue reservation.
