# Supplementary material for: A cross-cultural study of unwillingness to consume insects in Croatia, Lithuania, Portugal, Romania, and Mexico
Source: Front Nutr. 2025 Dec 8;12:1699378. doi: 10.3389/fnut.2025.1699378 (PMC12722814; doi:10.3389/fnut.2025.1699378)
Supplement: Supplementary file 1 [file Table_1.DOCX]

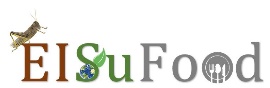
**ANKETA O JESTIVIM KUKCIMA**

Ovo prikupljanje podataka ima za cilj istražiti stavove potrošača i njihovo znanje o konzumaciji insekata u ljudskoj prehrani.​

Anketa se provodi u okviru projekta EISuFood, koji se istovremeno odvija u 18 zemalja (koordinatorice: Raquel Guiné, Portugal i Marijana Matek, Hrvatska).​

Etička načela se strogo poštuju; sudjelovanje je dobrovoljno, a svi prikupljeni podaci strogo su povjerljivi. Samo punoljetni sudionici koji daju svoj pristanak mogu ispuniti upitnik.

Unaprijed zahvaljujemo na vašoj suradnji.

​Imam 18 godina ili više i slažem se sudjelovati u anketi.

1. **Demografski podaci**​
2. **Dob:**       godina
3. **Spol:**

Ženski _1_ Muški _2_ Ne želim odgovoriti _3_

1. **​Obrazovni nivo:**

Postdiplomsko obrazovanje (magisterij ili doktorat)  _1_

Završeni sveučilišni studij  _2_

Bez završenog sveučilišnog studija  _3_

Ako nemate sveučilišnu diplomu, koliko ste godina išli u školu?:_____________3.a_

1. **Životno okruženje:**

Ruralno _1_ Urbano  _2_ Prigradsko  _3_

1. **Kućni dohodak u odnosu na prosjek u vašoj zemlji:**

Znatno niži  _1_ Niži  _2_ Jednak prosjeku  _3_ Viši _4_ Znatno viši _5_

1. **Karakterizacija navika sudionika**
2. **Jeste li ikada konzumirali insekte u obliku kulinarskih pripravaka, grickalica ili drugih proizvoda?**

Da  _1_ Ne  _2_ Ne znam/ Ne sjećam se  _3_

1. **Što vam pada na pamet kada čujete za jestive insekte? Molimo navedite do 5 riječi ili kratkih izraza koje povezujete s jestivim insektima.**

**1)______________________________________________________**

**2)______________________________________________________**

**3)______________________________________________________**

**4)______________________________________________________**

**5)______________________________________________________**

Zahvaljujemo na suradnji.
