# Supplementary material for: A cross-cultural study of unwillingness to consume insects in Croatia, Lithuania, Portugal, Romania, and Mexico
Source: Front Nutr. 2025 Dec 8;12:1699378. doi: 10.3389/fnut.2025.1699378 (PMC12722814; doi:10.3389/fnut.2025.1699378)
Supplement: Supplementary file 2 [file Table_2.DOCX]

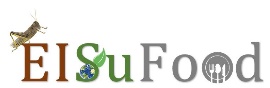
**EDIBLE INSECTS SURVEY**

This data collection is intended to investigate consumer perspectives and knowledge about insects for human consumption.

It is developed within the scope of the EISuFood project taking place in 18 countries simultaneously (Coordinator: Raquel Guiné, Portugal).

Ethical principles are strictly followed, participation is voluntary and all information collected is strictly confidential. Only adult participants, who give consent, respond to the questionnaire.

Thank you in advance for your cooperation.

We thank you in advance for your collaboration.

I am 18 years old or over and I agree to participate in the survey.

1. **Demographic Data**
2. **Age:**       years
3. **Sex:**

Female _1_ Male _2_ Don’t want to answer _3_

1. **Education level:**

Post-graduate education (master or doctorate) _1_

Completed a university degree _2_

No university degree  _3_

If you do not have a university degree, how many school years you have:_____________3.a_

1. **Living environment:**

Rural _1_ Urban  _2_ Suburban  _3_

1. **Household income in relation to the average in your country:**

Much lower  _1_ Lower  _2_ Equal to the average  _3_ Higher _4_ Much higher _5_

1. **Characterization of participants’ habits**
2. **Have you ever consumed insects as culinary preparations, snacks, or other derived products?**

Yes  _1_ No  _2_ Don´t know/Don´t remember  _3_

1. **What comes to your mind when you hear about edible insects? Please use up to 5 words or small expressions that you associate with edible insects.**

**1)______________________________________________________**

**2)______________________________________________________**

**3)______________________________________________________**

**4)______________________________________________________**

**5)______________________________________________________**

Thank you for your collaboration.

**The following survey was conducted exclusively in Mexico.**

**Acceptability of edible insects in different regions of Mexico**

**Section 1/4 Privacy policy**

IMPORTANT INFORMATION FOR THE PARTICIPANT

Purpose of the study: this study is designed to know the consumption habits of protein obtained from insects, and the differences between groups and segments of the Mexican population.

Main procedures of the study: the methodology for obtaining information is a survey-type instrument that contains at least 3 questions. The instrument was designed to be answered through an anonymous online link. To enter the form, you must click the box “I have read and accept the privacy policies” and then click accept to start the survey.

Duration of participation: you will be asked to participate in a session by completing a survey that will last no more than 5 minutes.

Significant Risks: there are no known risks associated with this project that are greater than those typically encountered in everyday life.

Potential benefits: there are no direct benefits to you, only for society. This study may help researchers learn more about food ingredient preferences and may help future researchers produce more attractive food ingredients for the population.

Compensation: none. You will not receive any payment for participating in this study.

Exclusion of participants: only people born in Mexico and with current residence in the same country may participate.

Context information

You are invited to participate in a research study to understand the perceptions and consumption preferences of protein obtained from insects. We ask that you read this form and ask any questions or concerns that may arise before agreeing to participate in the study. Your participation is voluntary.

This study is being carried out by: Dr. Cristina Chuck-Tecnológico de Monterrey, Dr. Lucio Rodríguez-UAdeC, Dr. César Ozuna-UG, Dr. Rosa María Mariscal-UIA, Dr. Anayansi Escalante-Aburto-UDEM.

Confidentiality

The study does not require you to provide personal information, such as your name. Your information will be treated confidentially to the extent permitted by technology. When the study is complete and the data has been analyzed, this information will be destroyed. Your responses will be used in a report containing only aggregates. The research team works to ensure confidentiality to the degree permitted by technology. It is possible, though unlikely, that unauthorized persons could access your responses because you are responding online.

Voluntary nature of the study

Your participation in this research is voluntary. There is no penalty for refusing to participate, and you are free to withdraw your consent and participation in this project at any time.

Contacts and questions

The team of researchers mentioned above has reviewed this questionnaire. For any questions, contact Dr. Cristina Chuck at: [cristina.chuck@tec.mx](mailto:cristina.chuck@tec.mx)

You may save or print a copy of this information for your records.

Declaration of consent

I have read the above information. I have had the opportunity to make questions and receive answers to my questions. I give my consent to participate in the study. This statement will be stored online if you choose to participate.

If you agree to participate in this research, please complete the survey. Thank you for your participation.

A copy of this privacy policy can be found here: <http://bit.ly/36B3jaa>

Question

I have read and accept the privacy policy

**Section 2/4 Acceptability of edible insects in different regions of Mexico**

1. **Have you ever consumed insects?**
2. Yes
3. No

**Section 3/4 Due to that you have not consumed insects previously,**

1. **Of the following reasons, which would justify why you do not include insects in your food/diet? (Select all that apply)**
2. I don’t like the idea
3. I don’t think they are tasty
4. Insects are not safe for to eat
5. The texture seems unpleasant to me
6. Just thinking about it makes me sick
7. Insects are dirty
8. The color does not look nice
9. I don’t want insect parts in my food
10. Other
11. **Of the following reasons, which would justify including insects as part of your nourishment/diet? (select all that apply)**
12. Lower environmental impact
13. The health benefits
14. Better price compared to other sources of protein
15. Greater availability in supermarkets and other shops
16. Companies certified in safety and quality
17. Improvement in texture and flavor aspects of insects-based products
18. Nothing would make me integrate insects into my diet
19. More information on how to prepare them
20. If insects do not add flavor or texture to the food
21. Other

**Section 4/4 Thanks for your participation!**
