# Supplementary material for: A cross-cultural study of unwillingness to consume insects in Croatia, Lithuania, Portugal, Romania, and Mexico
Source: Front Nutr. 2025 Dec 8;12:1699378. doi: 10.3389/fnut.2025.1699378 (PMC12722814; doi:10.3389/fnut.2025.1699378)
Supplement: Supplementary file 4 [file Table_4.DOCX]

**Aceptabilidad de insectos comestibles en diferentes regiones de México**

**Sección 1/4 Política de privacidad**

INFORMACIÓN IMPORTANTE PARA EL PARTICIPANTE

Propósito del estudio: Este estudio tiene como objetivo conocer los hábitos de consumo de proteínas obtenidas a partir de insectos, así como identificar las diferencias entre diversos grupos y segmentos de la población mexicana.​

Procedimientos principales del estudio: La metodología empleada para recopilar la información consiste en un instrumento tipo encuesta que contiene al menos tres preguntas. Este instrumento fue diseñado para ser respondido a través de un enlace en línea de forma anónima. Para acceder al formulario, es necesario marcar la casilla "He leído y acepto las políticas de privacidad" y luego hacer clic en "Aceptar" para iniciar la encuesta.

​Duración de la participación: Se le solicitará que participe completando una encuesta que tomará aproximadamente 5 minutos.​

Riesgos significativos: No se conocen riesgos asociados con este proyecto que sean mayores a los que se encuentran típicamente en la vida cotidiana.​

Beneficios potenciales: No hay beneficios directos para usted; sin embargo, este estudio puede contribuir a que los investigadores comprendan mejor las preferencias alimentarias, lo que podría ayudar en el desarrollo de ingredientes alimentarios más atractivos para la población en el futuro.​

Compensación: No se proporcionará ninguna compensación económica por participar en este estudio.​

Criterios de exclusión: Solo pueden participar personas nacidas en México y que residan actualmente en el país.​

Contexto de la información

Está invitado a participar en un estudio de investigación que tiene como objetivo comprender las percepciones y preferencias de consumo de proteínas obtenidas a partir de insectos. Le solicitamos que lea este formulario detenidamente y plantee cualquier pregunta o inquietud que pueda surgir antes de aceptar participar en el estudio. Su participación es completamente voluntaria.

Este studio es realizado por: Dra. Cristina Chuck-Tecnológico de Monterrey, Dr. Lucio Rodríguez-UAdeC, Dr. César Ozuna-UG, Dra. Rosa María Mariscal-UIA, Dra. Anayansi Escalante-Aburto-UDEM.

Confidencialidad

Este estudio no requiere que proporcione información personal, como su nombre. Sus respuestas serán tratadas con confidencialidad en la medida que la tecnología lo permita. Una vez que el estudio concluya y los datos hayan sido analizados, esta información será eliminada. Sus respuestas se utilizarán únicamente en informes que presenten datos agregados. El equipo de investigación se compromete a garantizar la confidencialidad de la información, dentro de las posibilidades que ofrece la tecnología. Sin embargo, dado que la encuesta se realiza en línea, existe una posibilidad, aunque poco probable, de que personas no autorizadas puedan acceder a sus respuestas.

Naturaleza voluntaria del estudio

**Su participación en esta investigación es voluntaria.** No existe ninguna penalización por negarse a participar, y usted tiene la libertad de retirar su consentimiento y abandonar este proyecto en cualquier momento.

Contacto y preguntas

El equipo de investigadores mencionado anteriormente ha revisado este cuestionario. Para cualquier pregunta, comuníquese con la Dra. Cristina Chuck al correo electrónico: cristina.chuck@tec.mx

Puede guardar o imprimir una copia de esta información para sus registros

Declaración de consentimiento

He leído la información anterior. He tenido la oportunidad de hacer preguntas y recibir respuestas a mis inquietudes. Doy mi consentimiento para participar en el estudio. Esta declaración se almacenará en línea si decide participar.

​**Si acepta participar en esta investigación, por favor complete la encuesta. Gracias por su participación.**

Si está de acuerdo en participar en esta investigación, por favor complete la encuesta. Gracias por su participación.

Una copia de la política de privacidad se encuentra aquí: <http://bit.ly/36B3jaa>

Preguntas

He leído y aceptado la política de privacidad

**Sección 2/4 Aceptabilidad de insectos comestibles en México**

1. **¿Alguna vez ha consumido insectos?**
2. Sí
3. No

**Sección 3/4 Debido a que no ha consumido insectos previamente,**

1. **De las siguientes razones, ¿Cuáles justificarían el por qué no ha incluido insectos en su alimento/dieta)? (Seleccione todas las que apliquen)**
2. No me gusta la idea
3. No creo que sean sabrosos
4. Los insectos no son seguros para consumo
5. La textura me parece desagradable
6. Sólo pensar en ello me enferma
7. Los insectos son sucios
8. El color no se ve agradable
9. No quiero partes de insectos en mi comida
10. Otro
11. **De las siguientes razones, ¿Cuáles justificarían incluir insectos como parte de su alimento/dieta? (Seleccione todas las que apliquen)**
12. Bajo impacto ambiental
13. Beneficios a la salud
14. Mejores precios comparados con otras Fuentes de proteína
15. Mayor disponibilidad en supermercados y otras tiendas
16. Compañías certificadas en seguridad y calidad
17. Mejora en aspectos de textura y sabor de los productos hechos a base de insectos
18. Nada me haría integrar insectos a mi dieta
19. Mayor información en cómo prepararlos
20. Si los insectos no añaden sabor o textura a la comida
21. Otro

**Sección 4/4 Gracias por su participación!**
