# Supplementary material for: A cross-cultural study of unwillingness to consume insects in Croatia, Lithuania, Portugal, Romania, and Mexico
Source: Front Nutr. 2025 Dec 8;12:1699378. doi: 10.3389/fnut.2025.1699378 (PMC12722814; doi:10.3389/fnut.2025.1699378)
Supplement: Supplementary file 5 [file Table_5.DOCX]

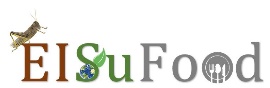
 **​VALGOMŲJŲ VABZDŽIŲ APKLAUSA**​

Šis duomenų rinkimas skirtas ištirti vartotojų požiūrį ir žinias apie vabzdžius, naudojamus maistui.

Jis vykdomas EISuFood projekto, vykstančio 18 šalių tuo pačiu metu (koordinatorė: Raquel Guiné, Portugalija. Elena Bartkiene, Lietuva), rėmuose.

Griežtai laikomasi etikos principų, dalyvavimas yra savanoriškas, o visi surinkti duomenys yra griežtai konfidencialūs. Į klausimyną atsako tik suaugę dalyviai, davę sutikimą.

Iš anksto dėkojame už jūsų bendradarbiavimą.

Aš esu 18 metų ar vyresnis ir sutinku dalyvauti apklausoje.

1. ​**Demografiniai duomenys**
2. **Amžius:**       metai
3. **Lytis:**

Vyras _1_ Moteris _2_ Nenoriu atsakyti _3_

1. **Išsilavinimo lygis:**

Aukštesnysis išsilavinimas (magistras ar daktaras)  _1_

Baigtas universitetinis išsilavinimas  _2_

Neturite universitetinio išsilavinimo  _3_

Jei neturite universitetinio išsilavinimo, kiek metų mokyklos lankėte?_____________3.a_

1. **Gyvenamoji aplinka:**

Kaimo  _1_ Miesto  _2_ Priemiestis  _3_

1. **Namų ūkio pajamos, palyginti su vidutinėmis jūsų šalyje:**

Gerokai mažesnės  _1_ Mažesnės  _2_ Lygu vidurkiui  _3_ Didesnės _4_ Gerokai didesnės _5_

1. ​**Dalyvių įpročių charakteristika**
2. **Ar kada nors vartojote vabzdžius kaip kulinarinius patiekalus, užkandžius ar kitus iš jų pagamintus produktus?**

Taip  _1_ Ne  _2_ Nežinau / Nepamenu  _3_

1. **Ką jums primena valgomi vabzdžiai? Prašome įrašyti iki 5 žodžių ar trumpų frazių, kurias siejate su valgomais vabzdžiais.**

**1)______________________________________________________**

**2)______________________________________________________**

**3)______________________________________________________**

**4)______________________________________________________**

**5)______________________________________________________**

Dėkoju už jūsų bendradarbiavimą.
