# Supplementary material for: A cross-cultural study of unwillingness to consume insects in Croatia, Lithuania, Portugal, Romania, and Mexico
Source: Front Nutr. 2025 Dec 8;12:1699378. doi: 10.3389/fnut.2025.1699378 (PMC12722814; doi:10.3389/fnut.2025.1699378)
Supplement: Supplementary file 6 [file Table_6.DOCX]

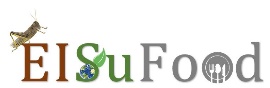
 **Pesquisa sobre insetos comestíveis**

Esta pesquisa coleta dados para realizar uma investigação sobre a perspetiva e o conhecimento do consumidor sobre insetos para consumo humano.

Este trabalho desenvolve-se no âmbito do projeto EISuFood que está a ser desenvolvido em 18 países em simultâneo (Coordenadora: Raquel Guiné, CERNAS-IPV, Portugal).

Os princípios éticos são rigorosamente seguidos, a participação é voluntária e todas as informações recolhidas são estritamente confidenciais. Apenas participantes adultos, que dão consentimento, têm permissão para responder ao questionário.

Agradecemos antecipadamente por sua colaboração.

Tenho 18 anos ou mais e concordo em responder.

1. **Dados demográficos**
2. **Idade:**       anos
3. **Sexo:**

Feminino _1_ Masculino _2_ Prefiro não indicar _3_

1. **Educação:**

Pós-graduação ou doutoramento  _1_

Com curso superior completo  _2_

Sem curso superior  _3_

Se não tem um curso superior, quantos anos de escolaridade tem?:_____________3.a_

1. **Meio onde vive:**

Rural _1_ Urbano  _2_ Suburbano  _3_

1. **Rendimento familiar em relação à média em Portugal:**

Muito inferior  _1_ Inferior  _2_ Igual á média  _3_ Acima da média _4_ Muito acima da média _5_

1. **Caracterização dos hábitos**
2. **Já comeu insetos como preparações culinárias, como snacks ou outros produtos derivados?**

Sim  _1_ Não  _2_ Não sei/Não me lembro  _3_

1. **O que vem à sua mente quando ouve falar de insetos comestíveis? Por favor, use até 5 palavras ou pequenas expressões que associa a insetos comestíveis:**

**1)______________________________________________________**

**2)______________________________________________________**

**3)______________________________________________________**

**4)______________________________________________________**

**5)______________________________________________________**

Obrigada pela colaboração.
