# Supplementary material for: A cross-cultural study of unwillingness to consume insects in Croatia, Lithuania, Portugal, Romania, and Mexico
Source: Front Nutr. 2025 Dec 8;12:1699378. doi: 10.3389/fnut.2025.1699378 (PMC12722814; doi:10.3389/fnut.2025.1699378)
Supplement: Supplementary file 7 [file Table_7.DOCX]

**
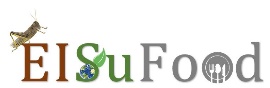
CHESTIONAR DESPRE INSECTELE COMESTIBILE**

Această colectare de date are scopul de a investiga perspectivele și cunoștințele consumatorilor despre insectele destinate consumului uman.

Este dezvoltată în cadrul proiectului EISuFood, desfășurat simultan în 18 țări (Coordonatori: Raquel Guiné, Portugalia & Monica Tarcea, România).

Principiile etice sunt respectate cu strictețe, participarea este voluntară și toate informațiile colectate sunt strict confidențiale. Doar participanții adulți, care își dau consimțământul, răspund la chestionar.

Vă mulțumim anticipat pentru cooperare.

Am 18 ani sau mai mult și sunt de acord să particip la sondaj.

1. **Date demografice**
2. **Vârstă:**       de ani
3. **Gen:**

Femeie _1_ Bărbat _2_ Nu doresc să răspund _3_

1. **Nivel de educație:**

Studii postuniversitare (master sau doctorat) _1_

Diplomă universitară obținută  _2_

Fără diplomă universitară  _3_

Dacă nu aveți o diplomă universitară, câți ani de școală ați finalizat?:_____________3.a_

1. **Mediul de viață:**

Rural _1_ Urban  _2_ Suburban  _3_

1. **Venitul gospodăriei în raport cu media națională:**

Mult mai mic  _1_ Mai mic  _2_ Egal cu media  _3_ Mai mare _4_ Mult mai mare _5_

1. **Caracterizarea obiceiurilor participanților:**
2. **Ați consumat vreodată insecte sub formă de preparate culinare, gustări sau alte produse derivate?**

Da  _1_ Nu  _2_ Nu știu / Nu-mi amintesc  _3_

1. **Ce vă vine în minte când auziți despre insectele comestibile? Vă rugăm să folosiți până la 5 cuvinte sau expresii scurte pe care le asociați cu insectele comestibile.**

**1)______________________________________________________**

**2)______________________________________________________**

**3)______________________________________________________**

**4)______________________________________________________**

**5)______________________________________________________**

Vă mulțumim pentru colaborare
